# Lower butyrylcholinesterase is associated with postoperative delirium in adult cardiac surgery patients: a secondary analysis of two prospective studies

**DOI:** 10.1186/s12871-026-03662-w

**Published:** 2026-02-10

**Authors:** L. P. Beyer, L. von zur Gathen, B. El-Rayah, M. Wolke, O. Dewald, S. T. Schaefer, U. Guenther

**Affiliations:** 1 Fakultät VI – Medizin und Gesundheitswissenschaften, Carl von Ossietzky Universität, Oldenburg, Germany; 2 Klinik für Innere Medizin, Ammerland-Klinik, Westerstede, Germany; 3 Klinik für Anästhesie und interdisziplinäre Intensivmedizin, Pius-Hospital Oldenburg, Oldenburg, Germany; 4 Universitätsklinik für Anästhesiologie, Intensivmedizin, Notfallmedizin, Schmerztherapie. Klinikum Oldenburg AöR, Oldenburg, Germany; 5 Klinik für Anästhesiologie und Intensivmedizin, St. Joseph-Stift Hospital, Bremen, Germany; 6 Klinik für Herzchirurgie, Universitätsklinikum Erlangen, Erlangen, Germany; 7 Fakultät VI – Medizin und Gesundheitswissenschaften, Department für Versorgungsforschung, Big Data in der Medizin, Carl von Ossietzky Universität, Oldenburg, Germany; 8 University of Groningen, University Medical Center Groningen, Groningen, Netherlands

**Keywords:** Delirium, Cognitive impairment, Cholinesterase, BChE, BuChE, Postoperative delirium, POD, Preoperative risk assessment

## Abstract

**Background:**

Postoperative delirium (POD) is a serious complication after surgery and is associated with prolonged stay in the Intensive-Care-Unit (ICU), increased mortality, and cognitive decline. Older patients undergoing cardiac surgery are at higher risk for POD. Disbalance in the cholinergic pathway activity has been suggested to play an important role in the pathogenesis of POD. This secondary analysis investigates the association between butyrylcholinesterase (BChE) activity and POD in adult cardiac surgery patients.

**Methods:**

A secondary analysis was performed using pooled data from two prospective studies including patients aged ≥ 50 years undergoing elective cardiac surgery. Preoperative cognitive baseline and BChE activity (preoperatively and on postoperative day one) were obtained as part of clinical routine care. POD was assessed by experienced clinicians using standardized diagnostic criteria (CAM-ICU, DSM-V, and medical records) up to five days after surgery. Association between BChE and POD were analyzed using multivariable logistic regression. Predictors were screened in univariate analyses (*p* ≤ 0.25) and included in the model using backward elimination based on Akaike’s Information Criterion.

**Results:**

A total of 188 patients’ datasets were included. The overall incidence of POD was 21%. Patients who developed POD had significantly lower preoperative BChE activity than those who did not (median 6.30 kU/L [IQR 5.55, 7.25] vs. median 7.70 kU/L [IQR 6.70, 8.90]; *p* < 0.001; d = 0.847). In multivariable logistic regression, BChE activity was the only significant factor associated with POD (OR 0.65, CI 95%: 0.48–0.86; p_adjusted_ < 0.028). Other factors did not reach statistical significance in the multivariable analysis. After surgery, BChE activity drop was significant in both groups. The median absolute value remained lower in the POD group (5.10 vs. 4.90 kU/L), though statistically non-significant.

**Conclusions:**

This study confirmed that lower preoperative BChE activity is associated with higher odds of developing POD in adult patients undergoing cardiac surgery. These results suggest that BChE activity may identify patients at risk for POD. We suggest that a postoperative decrease in BChE activity is due to inflammatory processes.

**Trial registration:**

*“Low cholinesterase activity is a risk factor for delirium after cardiac surgery*”: German Clinical Trials Registry (DRKS 00017144, registered 15.05.2019).

**Supplementary Information:**

The online version contains supplementary material available at 10.1186/s12871-026-03662-w.

## Background

Postoperative delirium (POD) is a common and serious complication in Intensive-Care-Unit (ICU) patients, particularly among elderly patients [1–3]. POD is characterized by acute cognitive dysfunction, including confusion, disorientation, and fluctuations in attention and awareness [4]. It is associated with a wide range of adverse outcomes, including prolonged hospital stays, increased healthcare costs, early onset of dementia and higher mortality rates [5–10]. Pathophysiological models of delirium include degenerative alterations related to aging, oxidative stress, inflammatory processes, neuroendocrine and circadian dysregulations, which altogether flow into neurotransmitter dysregulation and network disconnectivity [11]. The Systems Integration Failure Hypothesis by Maldonado proposes that POD is caused by a combination of factors, such as alteration in neurotransmitter function, impairment of sensory processing, as well as disruption in the functional connectivity between brain regions [11]. All models are intertwined at various levels of their signal cascades, and most describe mechanisms associated with a deficiency of acetylcholine (ACh) in the brain [12]. Most importantly, a shortness of ACh in basal forebrain cholinergic centres has been shown to be associated with impaired memory function and inability to focus attention, two of the key features of delirium [13–15].

Since ACh is metabolized by cholinesterases (ChE), cholinesterase-inhibitors contribute to elevated concentration of ACh in the synaptic cleft. Indeed, administration of cholinesterase -inhibitors improves cognitive function in patients with dementia [16–18]. Administration of the cholinesterase -inhibitor rivastigmine was reported to prevent postoperative delirium in some cases with pre-existing impaired cognition [19]. The family of ChE consists mainly of the butyrylcholinesterase (BChE; Enzyme Commission (EC) 3.1.1.8, other names: plasma cholinesterase, pseudocholinesterase) and the acetylcholinesterase (AChE, EC 3.1.1.7) [20]. AChE is found primarily on the membranes of erythrocytes, in neuromuscular junctions and synapses. BChE is produced by the liver, excreted to the plasma and hydrolyses many choline-based esters in the blood. Of note, BChE has a long half-life of 10 to 14 days. In the clinical setting, BChE is commonly perceived as a liver function test, reflecting the capacity of the protein synthesis and is often referred to as the ‘non-specific’ cholinesterase. BChE activity, though, was already shown to be reduced during inflammatory processes and has been reported to be associated with serious postoperative complications, such as increased mortality in cardiovascular and trauma patients [21–24]. Chronic inflammatory processes as well as anticholinergic side effects of anesthetics were suspected to be involved in these observations [25, 26].

When analyzing the association between BChE and the risk of POD, it is critical to consider preoperative cognitive impairment as a confounding variable, as several studies have shown it to be an important predictor of delirium [27, 28]. Previous studies examining the association between BChE activity and delirium did not account for baseline cognitive status [29, 30]. To address this gap, preoperative cognitive impairment was assessed in our study population.

The objective of this secondary analysis was to test the hypothesis that lower BChE activity is associated with a higher risk of postoperative delirium in adult patients undergoing elective cardiac surgery.

## Methods

### Study design and participants

This study was designed as a secondary analysis of pooled data from the papers “*Disorientation as a delirium feature in non-intubated patients: development and evaluation of diagnostic accuracy of the ‘Confusion Assessment Method for Intermediate Care Unit’ (CAM-IMC) - a prospective cohort study”* and “*Low cholinesterase activity is a risk factor for delirium after cardiac surgery*” conducted at a tertiary care hospital in Oldenburg, Germany [31, 32]. Both studies included adult surgical patients of at least 50 years of age undergoing elective cardiac surgery. In this secondary analysis, we analyze all patients with complete pre- and postoperative BChE measurement and evaluation for POD. For more detailed inclusion criteria, please refer to the previous publications [31, 32]. The study adheres to the principles of the Declaration of Helsinki. The original studies were approved by the Ethics Committee of the Medical Faculty of the Carl von Ossietzky University Oldenburg, Germany (“Low cholinesterase activity is a risk factor for delirium after cardiac surgery”: No. 2019-001; “Disorientation as a delirium feature in non-intubated patients: development and evaluation of diagnostic accuracy of the ‘Confusion Assessment Method for Intermediate Care Unit’ (CAM-IMC) - a prospective cohort study”: No. 2020-020). All patients gave written informed consent prior to assessment. The STROBE (Strengthening the Reporting of Observational Studies in Epidemiology) statement was followed for this study [33].

### Data collection and measurements

In both studies, patients were approached prior to scheduled surgery to assess preoperative cognitive status using the Mini-Cog^®^ and to collect demographic data (age, sex, weight, height) [34]. Clinical data including, comorbidities, type of surgery, and laboratory parameters including BChE activity were obtained from medical records. Patients were considered as cognitively impaired with a Mini-Cog^®^ test result of two or less points.

### Measurement of BChE

Blood samples were collected one day prior to the scheduled day of surgery and on the first postoperative day as part of the clinical routine. Samples were generally collected between 6:00 and 8:00 a.m. BChE activity and other standard laboratory analyses were evaluated using a clinical chemistry analyzer system (Cobas 501; Roche Diagnostics GmbH, Mannheim, Germany). BChE activity was expressed as kilounits per liter (kU/L).

### Postoperative delirium

**Fig. 1:**
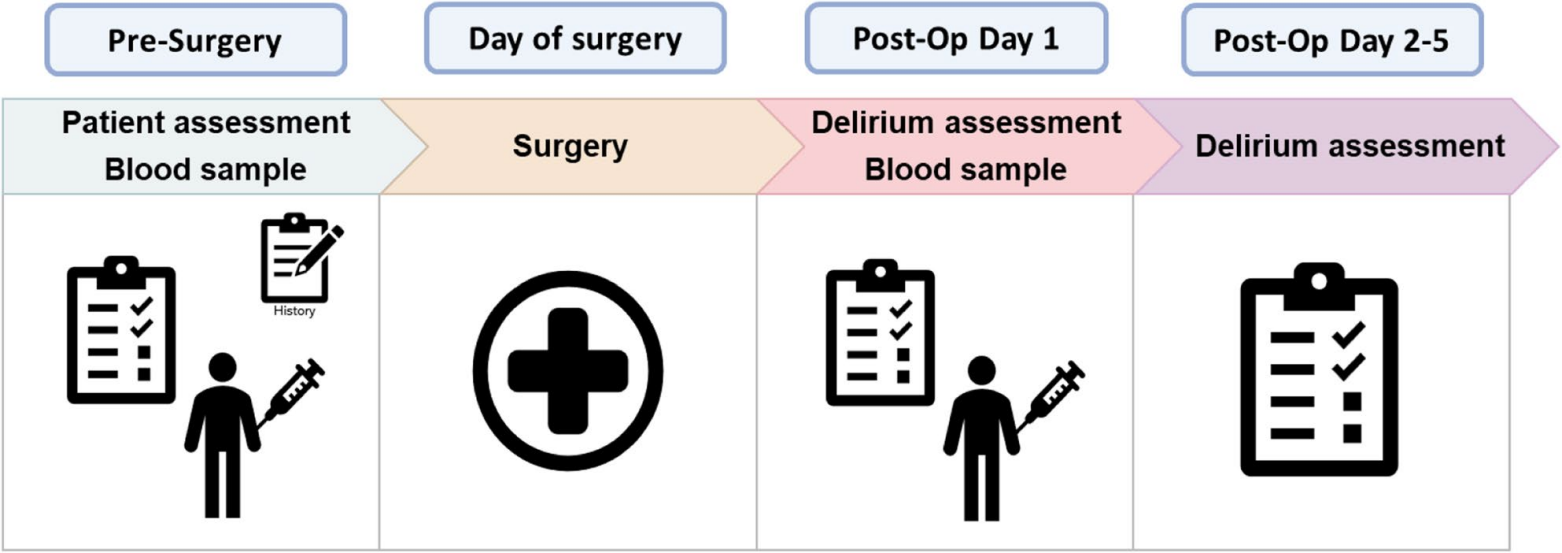
Patient assessment

POD was assessed independently of the clinical routine on the first postoperative day in both studies. The subsequent delirium assessment protocol varied among the studies with respect to the time of assessment. In the study “Disorientation as a delirium feature in non-intubated patients: development and evaluation of diagnostic accuracy of the ‘Confusion Assessment Method for Intermediate Care Unit’ (CAM-IMC) - a prospective cohort study”, delirium was assessed again on postoperative days two and three. In the study “*Low cholinesterase activity is a risk factor for delirium after cardiac surgery”*, delirium was further examined on postoperative day five. Assessments were performed by experienced clinicians based on standardized criteria (Diagnostic and Statistical Manual of Mental Disorders [DSM-V] criteria) and the medical documentation. The study assessment workflow is illustrated in Fig. 1.

### Statistical analysis

Patient demographics are presented as frequencies with percentages or medians with interquartile ranges. Depending on the scale and distribution, the Mann-Whitney U test (Wilcoxon rank sum test), chi-squared test, or Fisher’s exact test were used to test for differences between groups. The distribution of BChE activity (preoperative and postoperative) was tested using the Shapiro-Wilk test. Welch’s t-test was used to compare the means of BChE activity between pre- and postoperative groups. A paired t-test was used to examine the changes in BChE activity over time from preoperative to postoperative periods for both patients with and without POD. Effect sizes were reported using Cohen’s d. Multivariable binary logistic regression analysis was performed to identify significant independent risk factors associated with POD. Variables were first evaluated in a univariate logistic regression analysis. Variables associated with POD at a significance level of *p* < 0.25 in univariate analysis were subsequently included in the multivariable logistic regression model. The threshold was chosen to minimize risk of exclusion of potentially important predictors [35–38]. Multivariable model development was performed using a stepwise backward selection approach using the Akaike’s Information Criterion (AIC). Adjustments for multiple testing were made using the Bonferroni correction. Multicollinearity among covariates included in the finale multivariable logistic regression model was assessed using variance inflation factors (VIFs). VIF values < 5 were considered to indicate acceptable multicollinearity. Results are presented as Odds ratio (OR) with Confidence Intervals (CI) and p-values. The overall model was tested for significance using the Omnibus Test of Model Coefficients. Nagelkerke’s R^2^ was reported to judge the goodness of fit of the regression model. To verify if other factors contributed to the multivariable regression model, we recalculated the model after excluding preoperative BChE activity. To account for heterogeneity in POD assessment, a sensitivity analysis was performed in the subset of participants from the original study with POD evaluated until postoperative day five. The same multivariable logistic regression model used in the primary analysis was applied. Linear regression analyses were performed to explore correlations between BChE activity and age, CRP, and Mini-Cog^®^ results, respectively. Additionally, the area under the Receiver Operating Characteristic (AUROC) curve was calculated to evaluate the contribution of BChE activity to the prediction of POD. Receiver operating characteristic (ROC) curves for age, sex, Mini-Cog^®^ results, and BChE activity were plotted to evaluate their diagnostic performance in predicting POD. The Youden-Index was calculated to identify the optimal cut-off for BChE activity, and an additional cut-off was calculated to provide a sensitivity of 80%. Data was assessed regarding missing values for all variables. Cases with missing values in key predictors (pre- or postoperative BChE activity) or POD were excluded. Missingness for secondary variables were assumed to be missing completely at random. To address the risk of model overfitting an internal validation of the final multivariable logistic regression model was conducted using bootstrap resampling with 1000 replications.

A two-tailed p-value of < 0.05 was considered statistically significant. All p-values represent exploratory data analysis. A post-hoc power analysis was conducted for the primary endpoint using a two-sided Z-test with an alpha level of 0.05. Statistical analyses were performed using R Statistical language (version 4.2.2; R Core Team, 2022).

## Results

**Fig. 2:**
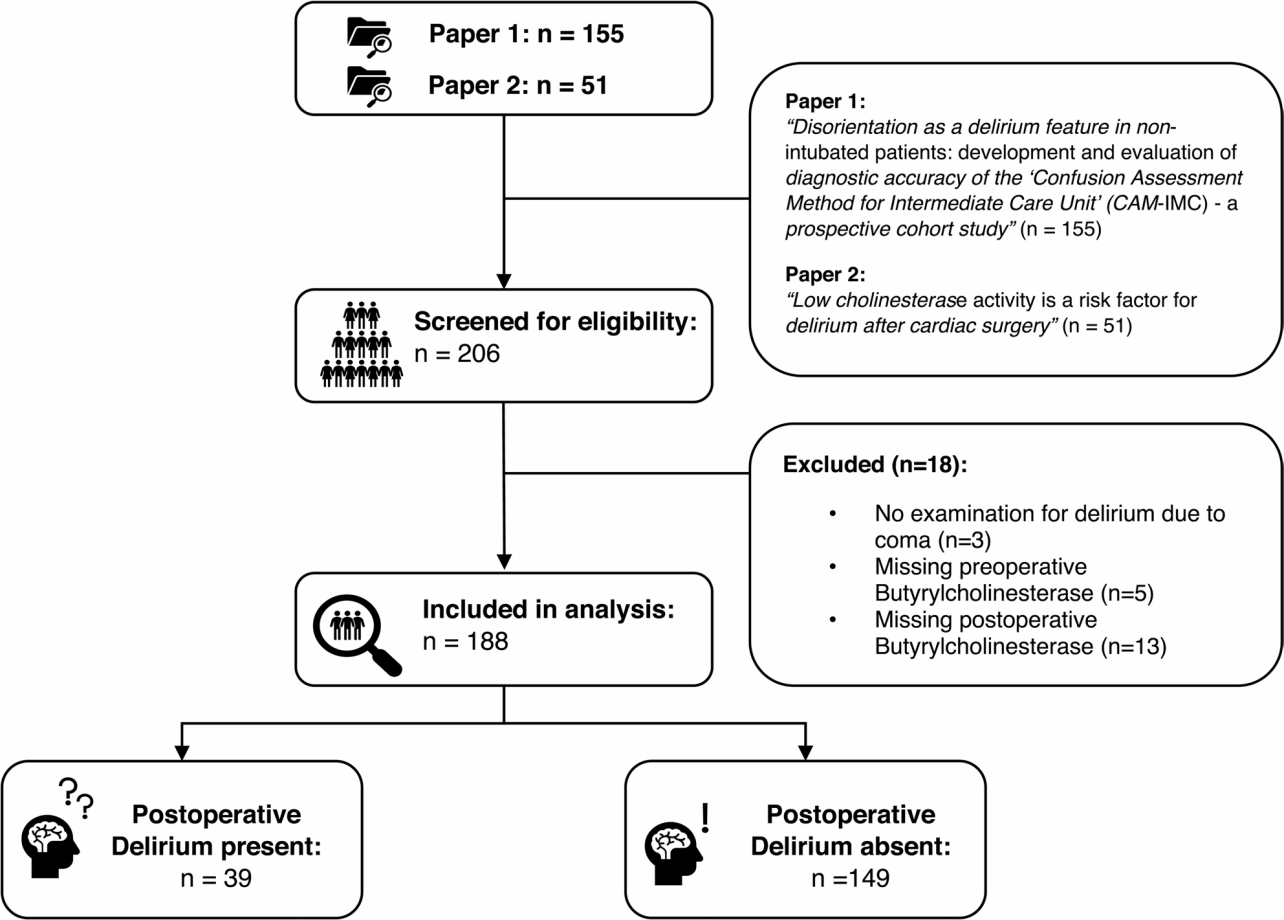
Patient flowchart

Of the 206 available patient datasets screened for eligibility, 188 patients with complete measurements for preoperative BChE, postoperative BChE, and POD assessment were included in this study (see Fig. 2). Eighteen patients were excluded due to lack of POD assessment, missing data for preoperative BChE activity or postoperative BChE activity. Secondary variables had twelve missing values for AST and two for bilirubin. The median age was 69 years (IQR 63, 75), and 42 patients (22%) were female.

Baseline characteristics are shown in Table 1. The overall incidence of POD was 21%. Patients experiencing POD were significantly older, and had lower levels of hemoglobin, creatinine, ALT, CRP and BChE. They were more often diagnosed with heart failure. Patients who underwent both coronary artery bypass graft (CABG) and aortic valve replacement at the same time had a significantly higher risk of POD, whereas patients who received CABG alone had a significantly lower risk of POD. For patients with preoperative cognitive impairment as measured by the Mini-Cog^®^ test, no significant difference was observed. Median BChE activity was 2.1 kU/l (IQR 0.6, 5.8) before surgery and 5.05 kU/L (IQR 4.40, 5.90) after surgery.

**Table 1:** Preoperative patients’ characteristics

Characteristics	Overall *n* = 188	No POD *n* = 149	POD *n* = 39	*p*-value	
Age (years), Median (IQR)	69 (63, 75)	67 (61, 73)	74 (71, 79)	**< 0.001**^**1**^	
Sex, n (%)				0.901^2^	
Female	42 (22%)	33 (22%)	9 (23%)		
Male	146 (78%)	116 (78%)	30 (77%)		
Weight (kg), Median (IQR)	86 (74, 96)	86 (75, 98)	80 (71, 94)	0.142^1^	
Height (cm), Median (IQR)	176 (170, 181)	176 (170, 182)	174 (166, 180)	0.200^1^	
BMI (kg/m^2^), Median (IQR)	27.5 (24.8, 31.0)	27.5 (25.0, 31.5)	27.3 (24.6, 29.6)	0.532^1^	
Leukocytes (10^9^/L), Median (IQR)	7.43 (6.59, 9.07)	7.44 (6.60, 9.08)	7.40 (6.25, 8.75)	0.909^1^	
Hemoglobin (g/dL), Median (IQR)	13.75 (12.50, 15.00)	13.90 (12.60, 15.10)	12.80 (11.55, 14.50)	**0.013**^**1**^	
Platelets (10^9^/L), Median (IQR)	234 (191, 276)	240 (202, 274)	192 (174, 288)	0.071^1^	
Creatinine (mg/dl), Median (IQR)	0.96 (0.84, 1.17)	0.93 (0.83, 1.15)	1.03 (0.92, 1.33)	**0.036**^**1**^	
AST (U/L), Median (IQR)	24 (19, 31)	24 (19, 31)	24 (20, 27)	0.783^1^	
ALT (U/L), Median (IQR)	23 (15, 32)	23 (15, 35)	17 (14, 29)	**0.047**^**1**^	
Bilirubin (mg/dL), Median (IQR)	0.50 (0.40, 0.70)	0.50 (0.40, 0.70)	0.60 (0.40, 0.80)	0.243^1^	
CRP (mg/L), Median (IQR)	2.1 (0.6, 5.8)	2.2 (0.7, 6.3)	1.1 (0.5, 4.1)	**0.045**^**1**^	
BChE (kU/L), Median (IQR)	7.35 (6.30, 8.63)	7.70 (6.70, 8.90)	6.20 (5.55, 7.15)	**< 0.001**^**1**^	
Heart failure, n (%)	69 (37%)	44 (30%)	25 (64%)	**< 0.001**^**2**^	
Chronic renal failure, n (%)	12 (6.4%)	7 (4.7%)	5 (13%)	0.132^3^	
Diabetes mellitus, n (%)	56 (30%)	45 (30%)	11 (28%)	0.808^2^	
Chronic pulmonary disease, n (%)	21 (11%)	15 (10%)	6 (15%)	0.392^3^	
Coronary-Arterial-Bypass-Graft, n (%)	120 (64%)	103 (69%)	17 (44%)	**0.003**^**2**^	
CABG and Aortic Valve Replacement, n (%)	37 (20%)	21 (14%)	16 (41%)	**< 0.001**^**2**^	
Mini-Cog^®^ Test, n (%)				0.350^2^	
No Cognitive Impairment	141 (75%)	114 (77%)	27 (69%)		
Cognitive impairment	47 (25%)	35 (23%)	12 (31%)		

^1^Wilcoxon rank sum test; ^2^Pearson’s Chi-squared test; ^3^Fisher’s exact test

*BChE* Butyrylcholinesterase, *BMI* Body mass index, *AST* Aspartate transaminase, *ALT* Alanine transaminase, *CRP* C-reactive-protein, *CABG* Coronary-Arterial-Bypass-Graft

**Fig. 3:**
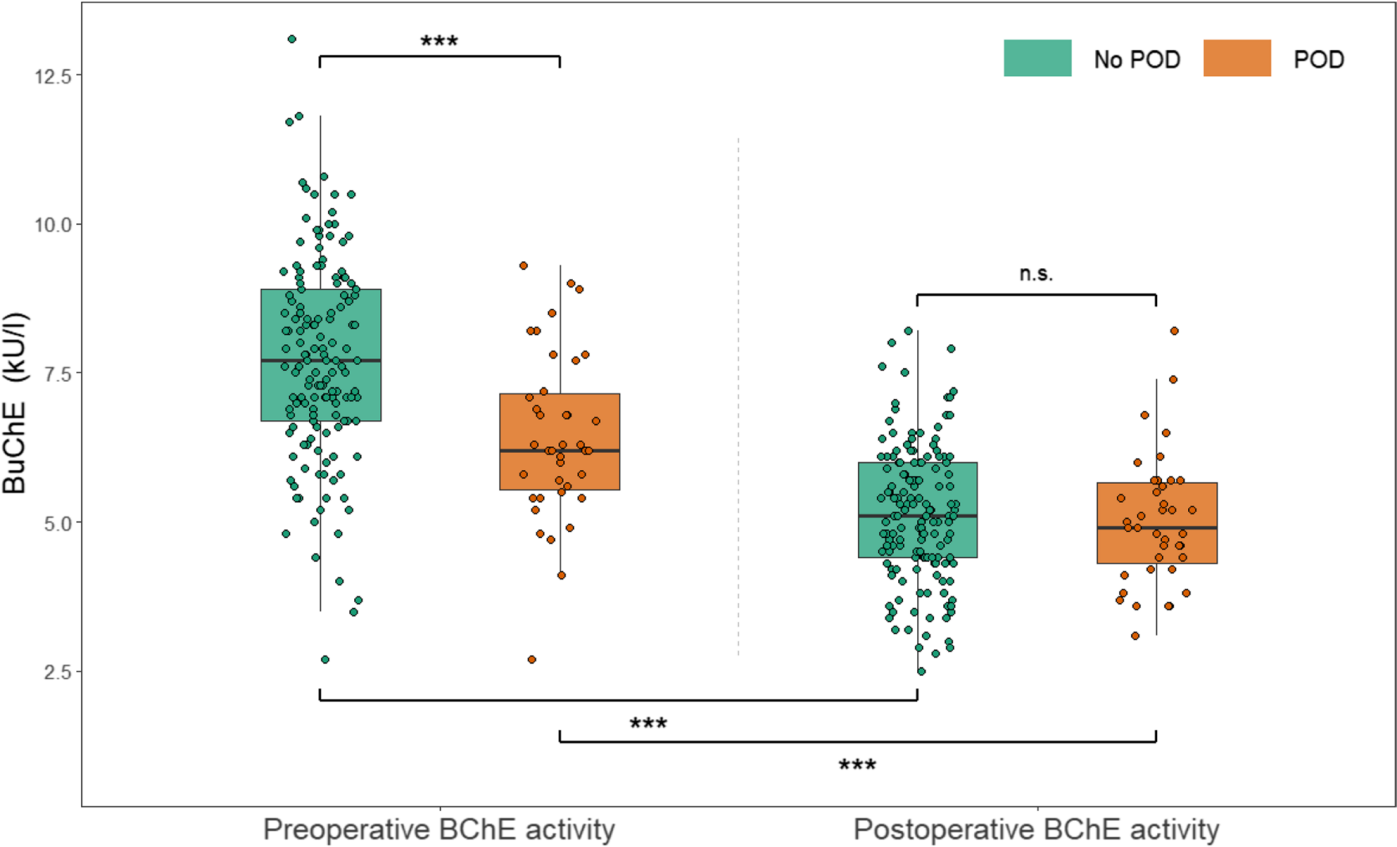
Preoperative and postoperative butyrylcholinesterase activity in patients with and without postoperative delirium. BChE = Butyrylcholinesterase; POD = Postoperative Delirium; *** = p-value < 0.001; n.s. = not significant

Patients who developed POD had significantly lower preoperative BChE activity compared to those without POD (median 6.20 kU/L [IQR 5.55, 7.15] vs. median 7.70 kU/L [IQR 6.70, 8.90]; *p* < 0.001; d = 0.847). Postoperatively, patients with POD showed slightly lower BChE activity compared to patients without POD, but without statistical significance (median 5.10 kU/L [IQR 4.40, 6.00] vs. median 4.90 kU/L [IQR 4.30, 5.65]; *p* < 0.586; d = 0.097). Both groups showed a significant decrease in BChE activity from pre- to postoperative measurements. Patients without POD experienced a decrease of 2.61 kU/L (mean; CI 95%: 2.44–2.78; *p* < 0.001; d = 2.479) from a median of 7.70 kU/L (IQR 6.70, 8.90) to a median of 5.10 kU/L (IQR 4.40, 6.00) and patients with POD showed a decrease of 1.41 kU/L (mean, CI 95%: 0.93–1.88, *p* < 0.001, d = 0.953) from 6.20 kU/L (median, IQR 5.55, 7.15) to 4.90 kU/L (median, IQR 4.30, 5.65). Figure 3 illustrates the difference in BChE activity between the two groups before and after surgery, and the development over time.

Factors associated with POD in univariate analyses included preoperative BChE activity, age, height, hemoglobin, creatinine, ALT, diagnosis of heart failure, chronic renal failure, CABG surgery, and combined CABG and aortic valve replacement surgery (see Appendix Table 1). Identified factors were entered into the initial multivariable logistic regression model. After stepwise backward selection only eight parameters (preoperative BChE activity, age, creatinine, ALT, CRP, diagnosis of heart failure and chronic renal failure and combined CABG and aortic valve replacement) remained in the final model (see Table 2). The model was statistically significant (χ^2^ [8] = 51.571; R^2^ = 0.375; *p* < 0.001). Using a Bonferroni correction for multiple testing, preoperative BChE activity was identified as the only significant individual factor associated with POD with an OR of 0.65 per of 1 kU/L increase (95% CI: 0.48–0.86; p_adjusted_ < 0.028). VIF analysis demonstrated low levels of multicollinearity among all covariates included in the adjusted logistic regression model. VIF values ranged from 1.06 to 1.47, indicating that multicollinearity was unlikely to bias coefficient estimates (see Appendix Table 5).

**Table 2:** Multivariable logistic regression of parameters associated with postoperative delirium

Characteristic	Coefficients	OR	95% CI	*p*-value	*p*_adjusted_^1^
BChE, preoperative (kU/L)	−0.43	0.65	0.48, 0.86	0.003	**0.028**
Age (years)	0.06	1.06	1.00, 1.13	0.062	0.499
Creatinine (mg/dl)	0.81	2.24	1.00, 6.16	0.080	0.639
ALT (U/L)	−0.03	0.97	0.93, 1.00	0.061	0.488
CRP (mg/L)	−0.02	0.98	0.94, 1.01	0.169	> 0.999
Diagnosis					
Heart failure	0.89	2.43	1.00, 5.93	0.049	0.390
Chronic renal failure	−1.4	0.25	0.04, 1.36	0.129	> 0.999
Operation					
Coronary-Arterial-Bypass-Graft and Aortic Valve Replacement	1.1	2.92	1.07, 7.97	0.035	0.279

^1^Bonferroni correction for multiple testing

*OR* Odds Ratio, *CI* Confidence Interval, *BChE* Butyrylcholinesterase, *ALT* Alanine transaminase, *CRP* C-reactive protein

After re-running the model without BChE activity as a predictor, no other variable contributed significantly to the prediction of POD (see Appendix Table 2). The overall model remained significant (χ^2^ [6] = 42.088; R^2^ = 0.314; *p* < 0.001). In the subset of participants with POD assessed until postoperative day 5 for sensitivity analysis (*n* = 51), the multivariable logistic regression model was statistically significant (χ² [8] = 29.64, R² = 0.597, *p* < 0.001). After Bonferroni adjustment for multiple testing, lower preoperative BChE activity remained the only significant predictor (see Appendix Table 3).

Both age (F(1, 186) = 13.87; R² = 0.069; *p* < 0.001) and CRP (F(1, 186) = 10.55; R² = 0.053; *p* = 0.001) were identified as significantly negatively correlated with BChE activity in linear regression analysis. For every additional year of age, BChE activity decreased by 0.055 kU/L in BChE activity. For every 1 mg/L increase in CRP, BChE activity decreased by 0.024 kU/L. No significant correlation was found between Mini-Cog^®^ results and BChE activity (F(1, 186) = 0.036; R² = 0.000; *p* = 0.850).

**Fig. 4:**
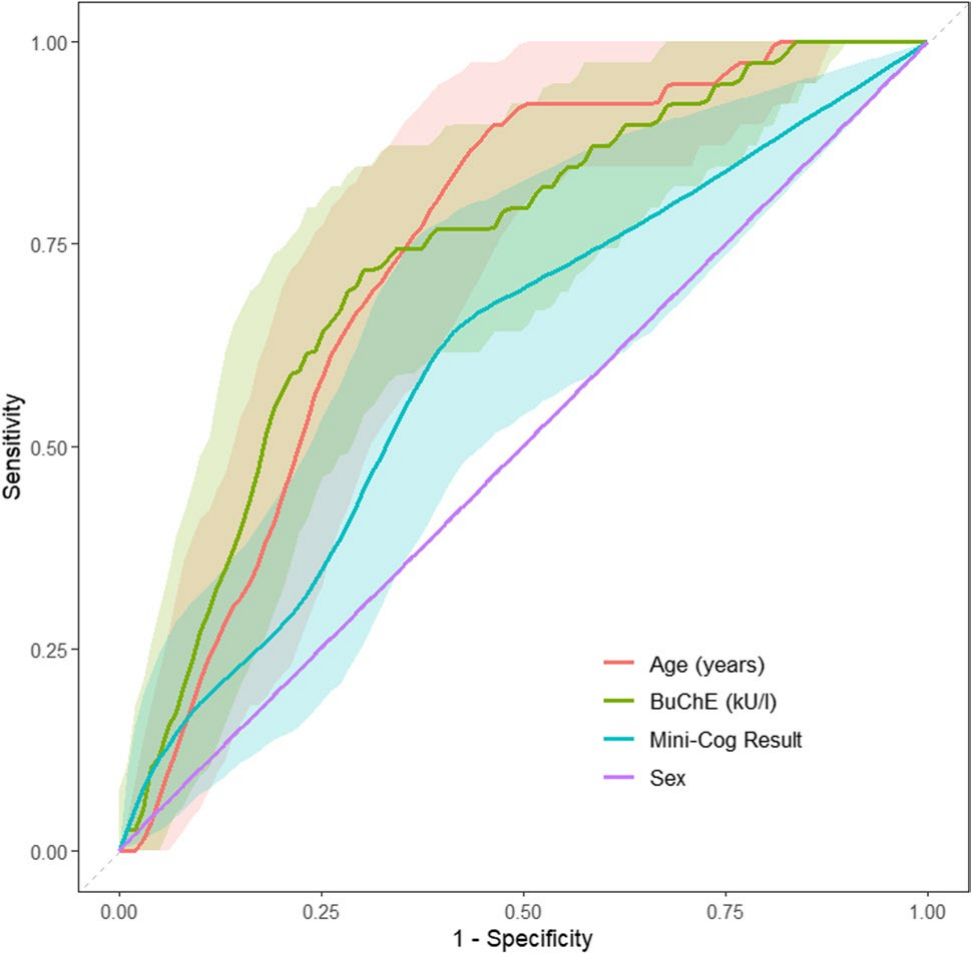
Receiver operating characteristic (ROC) analysis for age, butyrylcholinesterase (BChE), Mini-Cog^®^ result and sex for the outcome postoperative delirium. Shaded regions indicate 95% confidence intervals

The Area Under the Curve (AUC) for BChE was 0.734 (CI 95%: 0.65–0.82). The optimal cut-off point, determined using the Youden-Index, was 6.95 kU/L with a sensitivity of 72% and a specificity of 71%. When adjusting for a higher sensitivity threshold of 80%, the cut-off value increased to 7.75 kU/L, which resulted in a sensitivity of 79% and a specificity of 48%. Figure 4 presents the ROC analysis comparing age, sex, Mini-Cog^®^ result, and BChE activity in predicting POD, showing the diagnostic performance of each factor.

Internal validation demonstrated moderate discrimination and acceptable calibration of the final model, with an optimism-corrected c-index of approximately 0.74 and a calibration slope of 0.78, indicating limited overfitting. Bias-corrected explained variation was approximately 20%, consistent with stable model performance (see Appendix Table 4). A post-hoc power analysis indicated a statistical power of 83.3%.

## Discussion

This study confirmed that lower preoperative BChE activity is associated with an increased risk of developing POD in adult cardiac surgery patients. The odds of developing delirium increased by 35% for every 1 kU/L decrease in preoperative BChE activity, the optimal cut-off to predict POD by BChE activity was 6.95 kU/L. Out of 188 patients, 39 patients (21%) developed POD. BuChE activity declined postoperatively in both groups to nearly identical levels.

### Preoperative BChE as a risk factor of POD

BChE was the only significant independent factor in the regression model. After rerunning the model without BChE activity as a predictor for POD, the overall model remained significant, yet no other single predictor was independently significant. These results demonstrate BChE activity predicts significantly the development of POD, suggesting it could be a factor for the preoperative identification of patients at higher risk for POD. The optimal cut-off to predict POD by BChE activity in this study was 6.95 kU/L. Our findings are consistent with previous studies examining the relationship between BChE activity and POD [29, 39].

We found a decreased preoperative BChE activity to be correlated with higher age and increased CRP. The association between age and inflammation was shown earlier [27, 28]. For instance, depletion of ACh in the basal forebrain of a mouse model, mimicking early dementia-associated cholinergic loss, was associated with acute working memory deficits when bacterial lipopolysaccharide (LPS) was administered. Deleterious effects of LPS were prevented by the cholinesterase inhibitor donepezil. Furthermore, LPS had no effect when there was no prior ACh-deficiency [40, 41].

### Decline of postoperative BChE activity

We found a decrease in BChE activity after surgery to almost the same level in both groups with and without delirium. Similarly to our study, Cerejeira et al. reported even lower BChE activity after surgery in patients with POD [39]. Adam et al. also showed a significant decrease in BChE activity before and after surgery [30]. In line with our results, the study by John et al. and the CESARO trial confirmed a postoperative decrease in BChE activity but found no significant difference in activity between groups, either [29, 42]. Hughes et al. examined BChE activity in adults admitted to ICU with respiratory failure and/or shock [43]. While consecutive BChE activity did not show a significant association with delirium, though lower activity of BChE at the time of enrollment was associated with fewer days alive without delirium or coma and fewer days alive without delirium [43].

Many factors potentially responsible for this reduced activity have been described. For instance, opioids, which are administered regularly during ICU-therapy and cardiothoracic surgery, inhibit BChE in a selective way [44]. More recent results showed that the decline of the BChE activity is attributable to inflammatory processes. In line with these findings, BChE was negatively correlated with CRP. For instance, patients admitted acutely to the emergency department after trauma, showed decreasing enzyme activities during the following treatment period [24]. In septic patients, the same group of researchers showed that the level and the course of the BChE activities correlated with concentrations of biomarkers of systemic inflammation, such as Interleukin-6, Tumor-necrosis-factor-α and Procalcitonin [45]. In both works, sustained suppression of ChE activity was associated with a higher mortality [24, 45]. To shed more light on the relevance of ChE as a biomarker during ICU stay, more trajectories of BChE in patients with viral and non-viral sepsis in ICU were reported [46]. Patients had lower BChE activity even seven days after sepsis onset in patients with viral sepsis who died in the ICU [46]. These findings align with the concept of the cholinergic anti-inflammatory pathway, in which acetylcholine modulates systemic inflammation via vagal signaling [47, 48].

The underlying pathophysiological mechanisms of the quick postoperative decline in BChE activity remain unclear. Given the long half-life of 10 to 14 days, the rapid decrease in BChE is unlikely to be explained by reduced synthesis alone and may involve proteolysis, as shown earlier [49].

Cardiopulmonary bypass–related hemodilution also represents an important potential confounder, as it may contribute to an acute reduction in measured BChE activity. More generally, hemodilution following volume resuscitation (e.g., in sepsis) could partially explain postoperative decreases in enzyme activity. In our cohort, combined procedures involving coronary artery bypass grafting and aortic valve replacement were more frequent in patients who developed POD, reflecting greater surgical complexity, longer cardiopulmonary bypass and cross-clamp times, and increased intraoperative fluid administration. Additionally, impaired hepatic synthesis due to perioperative liver dysfunction may further contribute to reduced BChE activity.

### Limitations

Our study has several limitations that need to be considered. The nature of the secondary analysis limits the ability to establish causality between BChE activity and POD although it was adjusted for numerous confounders and multiple testing. Several clinically relevant confounders, including duration of surgery, cardiopulmonary bypass and cross-clamp times, perioperative exposure to sedatives, opioids or anticholinergic medications, and detailed inflammatory markers beyond CRP, were not collected in both original studies and therefore could not be included in the analysis. Given the established interactions between inflammation, cholinergic activity, and perioperative medication use, residual confounding cannot be excluded and should be considered when interpreting the findings.

The sample size was not powered for the hypothesis tested, which may limit the generalizability of the findings. The study included only patients undergoing cardiac surgery. This may limit the external validity of the findings to other surgical populations. In addition, the assessment duration differed between the two original studies. In one study, patients were screened for POD on postoperative days 1–3, whereas in the other study screening was performed on postoperative days 1 and 5. The incidence of POD varies over time, so inconsistent POD assessment may have led to misclassifications, especially in cases of late-onset delirium. According to the consensus-based guidelines, screening should be performed until the fifth postoperative day [50]. To address this limitation, a sensitivity analysis was conducted restricted to the patients with POD assessment until day 5 resulting in consistent results and confirmed robustness of the association between lower preoperative BChE activity and POD. To account for circadian fluctuations, blood samples were collected in the morning. This approach reflects the daily hospital routine and facilitated implementation in the routine care.

Although the number of postoperative delirium events was limited in relation to the number of predictors, several steps were taken to reduce the risk of overfitting. Model complexity was reduced through backward variable selection, internal validation using bootstrap resampling demonstrated only modest optimism in model performance, and Bonferroni adjustment was applied to reduce the risk of false-positive findings. While this conservative correction may have increased the risk of type II error for some covariates given the modest sample size, the persistence of the association between lower preoperative BChE activity and POD across sensitivity analyses and internal validation supports the robustness of this finding.

Well established risk factors of postoperative delirium, namely higher age and low pre-operative cognitive performance did not reach statistical significance in the final multivariable model. This study was not powered to detect the effect of age and preoperative cognition on POD occurrence. Since this research involved only scheduled cardiac surgery patients at moderate to higher age, pre-selected from their referring physicians for eligibility for cardiac surgery, a rather homogenous group was studied.

### Implementation and future research

Establishing preoperative BChE activity as a clinical screening tool for risk of POD in postoperative patients holds many challenges. Its implementation would increase the complexity and costs of preoperative testing, particularly in hospitals without on-site laboratory facilities capable of measuring BChE activity. In such settings, reliance on external laboratories and sample transportation would further increase costs and add logistical steps to the screening workflow. To avoid heterogenous interpretation and measurement of BChE activity, standardized testing procedures are essential. Such standardization is critical not only for future clinical implementation but also for the comparability of research findings.

Future research should focus on establishing a reliable standardized preoperative screening tool that could incorporate BChE activity to improve risk stratification of POD. In addition, further studies are needed on the perioperative dynamics of BChE with a particular focus on postoperative inflammatory processes, as BChE appears to act as a negative marker for systemic inflammation. A more comprehensive understanding of relevant interactions and potential confounding factors, as outlined in the “Limitations” section, is crucial.

## Conclusion

Our study found an association between lower preoperative BChE activity and the occurrence of POD in patients undergoing cardiac surgery. It was the only significant factor to increase risk for POD in the multivariable logistic regression model. BChE after surgery and in the context of intensive care should be interpreted as an inflammatory parameter rather than an indicator of hepatic synthesis. More research is needed to clarify BChE activity’s role in the cholinergic anti-inflammatory pathway and the possible role of ChE-inhibitors to modify inflammation and prevent delirium.

## Supplementary Information

Supplementary Material 1.

## Data Availability

The datasets used in the current study are available from the senior-author of original studies on reasonable request.

## Appendix

**Table 3:** Univariable logistic regression of parameters associated with postoperative delirium

**Characteristic**	**Coefficients**	**OR**	**95% CI**	**p‑value**
BChE, preoperative (kU/L)	-0.51	0.60	0.46, 0.76	**<0.001**
BChE, postoperative (kU/L)	-0.09	0.92	0.67, 1.25	0.597
Age (years)	0.11	1.12	1.06, 1.18	**<0.001**
Sex, male	-0.05	0.95	0.42, 2.30	0.901
Weight (kg)	-0.01	0.99	0.97, 1.01	0.375
Height (cm)	-0.03	0.97	0.94, 1.01	**0.149**
BMI (kg/m^2^)	-0.01	0.99	0.92, 1.06	0.718
Leukocytes (10^9^/L)	0.00	1.00	0.84, 1.17	0.962
Hemoglobin (g/dL)	-0.28	0.76	0.62, 0.93	**0.007**
Platelets‎ (10^9^/L)	0.00	1.00	0.99, 1.00	0.580
Creatinine (mg/dl)	0.93	2.53	1.32, 5.72	**0.013**
AST (U/L)	-0.01	0.99	0.95, 1.02	0.464
ALT (U/L)	-0.03	0.97	0.94, 1.00	**0.039**
Bilirubin (mg/dL)	0.46	1.58	0.63, 3.78	0.303
CRP (mg/L)	-0.01	0.99	0.95, 1.01	0.325
Diagnosis				
Heart failure	1.4	4.26	2.05, 9.16	**<0.001**
Chronic renal failure	1.1	2.98	0.84, 9.93	**0.076**
Diabetes mellitus	-0.10	0.91	0.40, 1.94	0.808
Chronic pulmonary disease	0.49	1.62	0.54, 4.34	0.352
Surgery procedure				
Coronary-Arterial-Bypass-Graft	-1.1	0.35	0.17, 0.71	**0.004**
Coronary-Arterial-Bypass-Graft and Aortic Valve Replacement	1.4	4.24	1.92, 9.36	**<0.001**
Mini-Cog® (< 3 Points)	0.37	1.45	0.65, 3.11	0.352

*OR* Odds Ratio, *CI* Confidence Interval, *BChE* Butyrylcholinesterase, *AST* Aspartate transaminase, *ALT* Alanine transaminase, *CRP* C-reactive-protein

**Table 4:** Multivariable logistic regression of parameters associated with postoperative delirium without butyrylcholinesterase as a predictor

**Characteristic**	**OR**	**95% CI**	**p-value**	p_adjusted_^1^
Age (years)	1.07	1.01, 1.14	0.018	0.110
Haemoglobin (g/dl)	0.84	0.66, 1.07	0.155	0.930
Creatinine (mg/dl)	1.76	0.87, 4.28	0.166	0.998
ALT (U/L)	0.98	0.94, 1.00	0.124	0.743
Diagnosis				
Heart failure	2.90	1.27, 6.78	0.012	0.073
Operation				
Coronary-Arterial-Bypass-Graft and Aortic Valve Replacement	2.45	0.98, 6.05	0.052	0.312

^1^Bonferroni correction for multiple testing

*OR* Odds Ratio, *CI* Confidence Interval, *ALT* Alanine transaminase

**Table 5:** Multivariable logistic regression of parameters associated with postoperative delirium in the subset of participants with POD assessed until postoperative day 5

**Characteristic**	**OR**	**95% CI**	**p‑value**	p_adjusted_^1^
BChE, preoperative (kU/L)	0.34	0.15, 0.62	0.002	0.018
Age (years)	0.95	0.83, 1.08	0.456	> 0.999
Creatinine (mg/dl)	3.18	1.07, 20.0	0.100	0.803
ALT (U/L)	0.91	0.84, 0.98	0.018	0.144
CRP (mg/L)	0.79	0.47, 1.12	0.266	> 0.999
Diagnosis				
Heart failure	2.68	0.39, 24.3	0.335	0.999
Chronic renal failure	0.04	0.00, 0.49	0.024	0.189
Operation				
Coronary-Arterial-Bypass-Graft and Aortic Valve Replacement	2.20	0.36, 16.6	0.400	> 0.999

^1^Bonferroni correction for multiple testing

*OR* Odds Ratio, *CI* Confidence Interval, *BChE* Butyrylcholinesterase, *ALT* Alanine transaminase, *CRP* C-reactive protein

**Table 6:** Internal validation of the final multivariable logistic regression model for postoperative delirium using bootstrap validation with 1000 resamples showing original model performance, optimism, and optimism-corrected estimated

**Metric**	**Original**	**Training**	**Test**	**Optimism**	**Optimism-corrected**	**n**
Dxy	0.58	0.64	0.54	0.10	0.48	1000
R²	0.30	0.35	0.25	0.10	0.20	1000
Intercept	0.00	0.00	−0.30	0.30	−0.30	1000
Slope	1.00	1.00	0.78	0.22	0.78	1000
Emax	0.00	0.00	0.12	0.12	0.12	1000
B	0.11	0.10	0.11	−0.02	0.12	1000

*Dxy* Somers’ D, *R*² Pseudo R^2^ , *B* Brier score

**Table 7:** Variance inflation factors for predictors included in the adjusted multivariable logistic regression model

**Variable**	**VIF**
BChE, preoperative (kU/L)	1.16
Age (years)	1.08
Creatinine (mg/dl)	1.30
ALT (U/L)	1.06
CRP	1.13
Diagnosis	
Heart failure	1.11
Chronic renal failure	1.47
Operation	
Coronary-Arterial-Bypass-Graft and Aortic Valve Replacement	1.21

*VIF* Variance inflation factor, *BChE* Butyrylcholinesterase, *ALT* Alanine transaminase, *CRP* C-reactive protein
